# Incidence of Deep Vein Thrombosis in Neurointensive Care Unit Patients—Does Prophylaxis Modality Make Any Difference?

**DOI:** 10.5005/jp-journals-10071-23111

**Published:** 2019-01

**Authors:** Shailaja S Behera, Mathangi Krishnakumar, Radhakrishnan Muthuchellappan, Mariamma Philip

**Affiliations:** 1 Department of Anesthesiology, Pain Medicine and Critical Care, All India Institute of Medical Sciences, Ansari Nagar, New Delhi, India; 2,3 Department of Neuroanaesthesia and Neurocritical Care Neurosciences Faculty Centre, NIMHANS, Bengaluru, Karnataka, India; 4 Department of Biostatistics, National Institute of Mental Health and Neurosciences, Hosur Road, Bengaluru, Karnataka, India

**Keywords:** Deep vein thrombosis, Heparin, Intracranial malignancy, Neurocritical care, Thromboprophylaxis

## Abstract

**Background and aims:**

To determine the incidence of upper and lower limb deep vein thrombosis (DVT) using ultrasonography (USG) in adult patients admitted to neuro-medical and neurosurgical intensive care unit (ICU).

**Materials and methods:**

In this prospective observational study, patients admitted to the medical and surgical neuro-ICU and remained in the ICU for more than 48 hours were recruited. All patients were clinically examined for DVT. Basilic and axillary veins in the upper limbs and popliteal and femoral veins in the lower limbs were screened for DVT using USG. USG examination was performed on the day of admission to ICU and thereafter every 3rd day till discharge from ICU or death. Intermittent pneumatic compression (IPC) stockings were applied to the lower limbs to all the patients in both ICUs. Unfractionated heparin (UFH) was given subcutaneously to neuromedical ICU patients, while in surgical ICU, it was left to the discretion of the neurosurgeons.

**Results:**

A total of 130 adult patients were admitted to the ICU during the 8 month study period. Thirty patients were excluded and the remaining 98 patients’ (38 in medical and 60 in surgical ICU) data were analyzed. None of the 38 medical ICU patients developed DVT, while in neurosurgical ICU, 4 out of 60 patients developed DVT.

**Conclusion:**

A combination of UFH and IPC stockings were effective in minimizing the DVT in neuromedical ICU patients. In surgical patients, through IPC stockings were effective, UFH can be considered for patients with intracranial malignancy.

**How to cite this article:**

Behera SS, Krishnakumar M, Muthuchellappan R, Philip M. Incidence of Deep Vein Thrombosis in Neurointensive Care Unit Patients—Does Prophylaxis Modality Make Any Difference? Indian Journal of Critical Care Medicine, January 2019;23(1):43-46.

## INTRODUCTION

Deep vein thrombosis (DVT) is a major preventable cause of morbidity and mortality in ICU patients. The reported incidence of DVT and pulmonary thromboembolism (PE) in untreated neurosurgical patients varies between 18 to 50% and 0 to 25%, respectively.^1^ Ten to thirty percent of medical and surgical intensive care unit patients develop DVT within the first week of intensive care unit admission.^2^ Patients admitted to neuro-ICU (both medical and surgical) have multiple risk factors which predispose them to develop DVT. These include immobility, intracranial malignancy, osmotic diuretic-induced dehydration, inherited thrombophilia (e.g., antiphospholipid syndrome), head and spine injuries and hyperestrogenic states (pregnancy, postpartum and oral contraceptive pills). It is difficult to estimate the risk precisely in this subset of the population because of a lack of standard definition for venous thromboembolism (VTE) (i.e., clinically symptomatic or asymptomatic) and variation in detection methods.^3^ VTE in neuro-ICU has unique implications compared to other settings. In the setting of both ischemic and hemorrhagic central nervous system (CNS) pathology, the otherwise routine treatment with anticoagulation takes on an entirely different risk (intracranial bleeding) *vs.* benefit (reduction in VTE incidence) balance. Sometimes, even if patients are at risk of developing VTE (e.g. traumatic brain injury), pharmacological thromboprophylaxis is not initiated due to fear of intracranial bleeding. In our study, we intended to evaluate the incidence of DVT in both the upper and lower limbs in patients admitted to neuro-ICU.

## MATERIALS AND METHODS

This prospective observational study was conducted throughout 8 months in a tertiary care 33 bedded neuromedical and neurosurgical ICU. Institute ethics committee approval was obtained before the beginning of the study. Written informed consent was obtained from eligible patients or their surrogates. Adult patients older than 18 years and who stayed in the ICU for more than 48 hours were included in this study. Pregnant women and patients with a diagnosis of DVT before/at ICU admission were excluded from the study.

At ICU admission, patients were clinically examined for features suggestive of DVT (swelling, tenderness and prominent collateral superficial veins) in both upper and lower limbs and this was continued until patient discharge from ICU or death. Risk stratification for deep vein thrombosis was done using Well's score.^4^ Bedside ultrasonography for DVT was done using a high-frequency linear probe (8–12 MHz, Logiqe, GE Medical Systems, Jiangsu, China and/or 6–13 MHz, TurboM, Fujifilm Sonosite, WA, USA) for both upper and lower limbs. In the lower limb saphenofemoral junction, femoral vein at the inguinal crease, the popliteal vein in popliteal fossa and posterior tibial and peroneal veins were assessed and in the upper limbs, basilic, cephalic and axillary veins were assessed. USG examination included compressibility, color flow, Doppler study, flow augmentation and presence of echogenic materials.^5^ When USG was positive for proximal vein DVT, transthoracic echocardiography was done to screen for pulmonary embolism. All the above examination was repeated every alternate day for first 7 days and then every third day after that in neurosurgery patients and in medical patients screening was done on the day of admission to ICU and later every third day till death or discharge.

All patients in both neuromedical and neurosurgical ICUs received intermittent pneumatic compression (IPC) stockings for lower limbs. It was a routine clinical practice to administer 5000 IU of unfractionated heparin (UFH) subcutaneously to patients in neuromedical ICU. In neurosurgical ICU, this was decided by the neurosurgeons.

### Statistical Analysis

Data were analyzed using IBM statistical package for the social sciences (SPSS) Statistics for Windows, Version 21.0 (Armonk, NY). Data were presented as mean and standard deviation for parametric data, median and interquartile ranges for ordinal data and as percentages for categorical variables. The differences parametric data were analyzed with student's t-test. A *p* value < 0.05 was taken to be significant.

**Flowchart 1:**
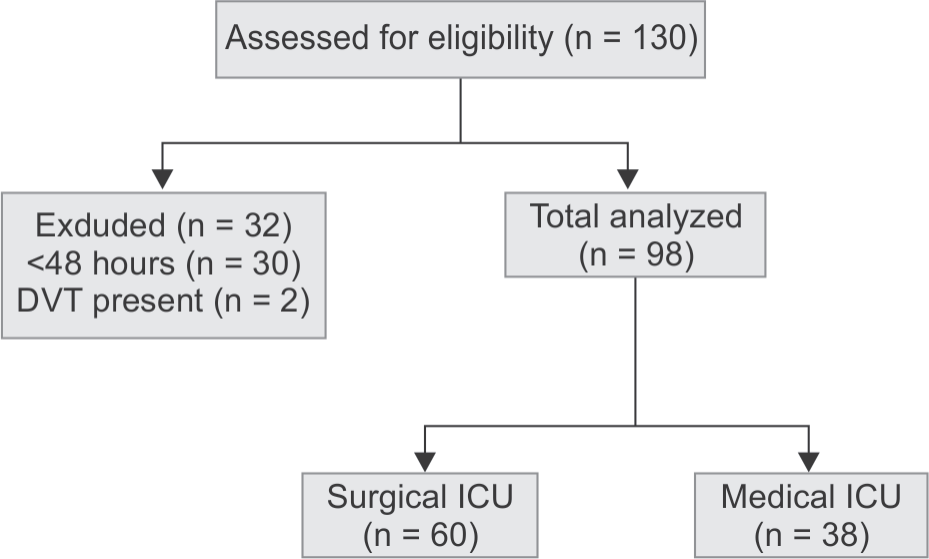
Consort diagram

## RESULTS

A total of 130 patients were admitted to the ICU during the study period. Thirty patients stayed less than 48 hours in the ICU, and two patients were diagnosed with DVT at the time of ICU admission. The remaining 98 patients’ data were included in the analysis (Flowchart 1). The demographics of the patient population are given in Table 1. Distribution of patient population based on diagnosis is given in Table 2. Patients in the medical ICU stayed significantly longer as compared to surgical patients. The median (interquartile range) Well's score in the medical and surgical ICU patients were 2.^1-3^ Thirtyfive patients in medical ICU received a combination of IPC stockings and UFH (5000 IU subcutaneous, twice daily) and the remaining three received only IPC stockings. In surgical ICU, all the 60 patients received only IPC stockings. None of the medical patients developed DVT based on ultrasound examination. In surgical patients, four patients (6.7%) developed DVT (Table 3). Out of the four patients, three developed DVT in the second week and the remaining one developed in the 5th week. All the four patients had proximal femoral vein DVT, and a TTE examination did not show evidence of pulmonary vein thrombus. These patients had swelling of the involved leg. All these patients were put on enoxaparin 60 mg subcutaneously twice daily. One of the patients died due to poor neurologic status.

**Table 1:** Demographics of patient population

		*Medical ICU (n = 38)*	*Surgical ICU (n = 60)*
Male: female ratio		21:17	45:15
Mean (SD) age in years	Male	36.9 ± 12	40.9 ± 15.2
	Female	41.4 ± 16.6	51 ± 15.4
Duration of ICU stay in days (mean ± SD)		24.8 ± 27.5	13.9 ± 10.3*

* p<0.05

**Table 2:** Distribution of patient population and diagnosis in medical and surgical ICU.

*Diagnosis (Medical) Number of patients (n = 38)*		*Diagnosis (Surgical) Number of patients (n = 60)*	
Guillain–Barre syndrome	16	TBI	34
Myasthenia gravis	9	Intracranial aneurysm	12
Stroke	5	Intracranial tumor	8
Cerebral venous sinus thrombosis	5	Spinal cord injury	4
Others	3	Others	2

**Table 3:** Details of patients (surgical) who developed DVT during ICU stay

*Patient no*	*Age years*	*Diagnosis*	*Duration of ICU stay (days)*	*DVT diagnosis at ICU day*	*DVT site (vein)*	*GCS at admission*	*GCS at discharge*	*Well's score*
1	19	Pontomedullary lesion	31	16	RCF	E1VtM4	Died	3
2	18	Posterior fossa lesion	65	37	RCF	E1VtM3	E1VtM5	2
3	57	Craniopharyngioma	27	10	LCF	E4V5M6	E4VtM6	2
4	58	Left frontal glioma	25	16	RCF	E4VtM5	E4VtM6	2

RCF: Right common femoral vein LCF: Left common femoral vein

## DISCUSSION

In this observational study, we observed a 6.7% incidence of DVT in surgical ICU patients while none in the medical ICU developed DVT.

The estimated risk of VTE in the ICU is around 20 per 1000 patients. The incidence of DVT in neuro-ICU is presumed to be higher with studies reporting an incidence of 18–50%.^1^ In a study by Henwood et al., which included 237 neurosurgical patients receiving UFH and pneumatic compression sleeves within 24 hours of admission, the incidence of DVT was around 9.7%.^6^ On the contrary, in our study, the incidence was around 6% in surgical patients without pharmacological prophylaxis. The low incidence could be attributed to patient ethnicity. In a meta-analysis, it was shown that the incidence of DVT is low in Asian surgical population even in the presence of high-risk factors.^7^ However, this study excluded neurosurgical patients. Earlier studies have documented DVT within the first week of neurosurgical intervention and suggested weekly ultrasound screening for DVT.^5,8^ But in these studies, heparin was initiated within 48 hours of surgical intervention. But in our study, we did ultrasound screening every third day as we did not use pharmacological prophylaxis in our patients and expected the DVT incidence to be high. On the contrary, we did not find an increased incidence of DVT, and moreover, the four patients who developed DVT was diagnosed in the second and fifth week of their ICU stay. Hence, it is better to perform an ultrasound examination weekly until patients stay in the ICU to pick up the delayed onset of DVT.

In the neurosurgical population, the highest risk for DVT is in patients with brain tumors (28–43%), followed by patients undergoing craniotomy (25%), and those with head injury (20%).^1^ Among brain tumors, patients with cerebral metastasis and glioma have the highest incidence of DVT.^9^ All the patients who were diagnosed with DVT in our study belonged to the intracranial tumor category. One reason for the increased incidence in this patient group in our study could be due to the lack of initiating pharmacological prophylaxis. Smith et al. in his cohort of 1148 patients with brain tumor, showed an incidence of 13.7% for DVT, 2.2% for heparin-induced hemorrhagic complications and 0.7% for hemorrhage induced neurological deficits. This shows that patients with high-grade primary brain tumors and metastatic lesions should receive aggressive preventative measures in the postoperative period as the DVT incidence outweighs the hemorrhagic complications.^10^ In spite of several population studies proving the efficacy of pharmacological therapy following neurosurgery, the reluctance and the fear of intracranial hemorrhage have been a major hindrance in the implementation of pharmacological therapy for DVT prevention at our institute. This is especially true after brain tumor surgery where there is a possibility of bleeding from the tumor bed site in the immediate postoperative period following near-total/partial excision. However, recent guidelines by the Neurosurgical society encourage the use of pharmacological prophylaxis in all neurosurgical patients within 24–48 hours of craniotomy with either UFH or low molecular weight heparin (LMWH).^3^ One must be cautious with LMWH as a meta-analysis has shown that the incidence of hemorrhagic complications was more with LMWH in the Asian population.^7^ All the four patients who developed DVT were treated with LMWH. This did not result in hemorrhagic complications as they were given between the 2nd week and 5th week after surgery during which the surgical site bleeding is minimal.

The present study had zero incidences of DVT in neuromedical ICU patients. This could have been due to the implementation of combined prophylaxis in this group of patients. In contrast to the surgical group, the neuromedical patients are mobilized earlier, have less activation of the inflammatory response and have a relatively lesser contraindication for the institution of early pharmacotherapy.

This study was limited by smaller sample size. This was also a single center study with no follow-up beyond the ICU stay. Further randomized controlled trials will help determine the efficacy of one method of prophylaxis over the other with keeping the risk-benefit balance in mind. Whether a blanket treatment or a tailor-made approach for DVT management of neurointensive care unit patients needs to be ascertained.

## CONCLUSION

A combination of UFH and IPC stockings were effective in minimizing the DVT in neuromedical ICU patients. In surgical patients, through IPC stockings were effective, UFH can be considered for patients with intracranial malignancy to reduce DVT.

## References

[1] Browd SR,, Ragel BT,, Davis GE,, Scott AM,, Skalabrin EJ,, Couldwell WT. (2004;). Prophylaxis for deep venous thrombosis in neurosurgery: a review of the literature.. Neurosurgical focus..

[2] Attia J,, Ray JG,, Cook DJ,, Douketis J,, Ginsberg JS,, Geerts WH. (2001;). Deep vein thrombosis and its prevention in critically ill adults.. Archives of internal medicine..

[3] Nyquist P,, Bautista C,, Jichici D,, Burns J,, Chhangani S,, DeFilippis M, (2016;). Prophylaxis of Venous Thrombosis in Neurocritical Care Patients: An Evidence-Based Guideline: A Statement for Healthcare Professionals from the Neurocritical Care Society.. Neurocritical care..

[4] Wells PS,, Owen C,, Doucette S,, Fergusson D,, Tran H. (2006;). Does this patient have deep vein thrombosis?. JAMA..

[5] Patel AP,, Koltz MT,, Sansur CA,, Gulati M,, Hamilton DK. (2013;). An analysis of deep vein thrombosis in 1277 consecutive neurosurgical patients undergoing routine weekly ultrasonography.. Journal of neurosurgery..

[6] Henwood PC,, Kennedy TM,, Thomson L,, Galanis T,, Tzanis GL,, Merli GJ, (2011;). The incidence of deep vein thrombosis detected byroutine surveillance ultrasound in neurosurgery patients receiving dual modality prophylaxis.. Journal of thrombosis and thrombolysis..

[7] Yeo DX,, Junnarkar S,, Balasubramaniam S,, Tan YP,, Low JK,, Woon W, (2015;). Incidence of venous thromboembolism and its pharmacological prophylaxis in Asian general surgery patients: a systematic review.. World journal of surgery..

[8] Khaldi A,, Helo N,, Schneck MJ,, Origitano TC. (2011;). Venous thromboembolism: deep venous thrombosis and pulmonary embolism in a neurosurgical population.. J of Neurosurg.

[9] Cote DJ,, Smith TR. (2016;). Venous thromboembolism in brain tumor patients.. Journal of clinical neuroscience:.

[10] Smith TR,, Nanney III AD,, Lall RR,, Graham RB,, McClendon Jr J,, Lall RR, (2015;). Development of venous thromboembolism (VTE) in patients undergoing surgery for brain tumors: results from a single center over a 10 year period.. Journal of Clinical Neuroscience..

